# Acute kidney injury in non-renal medical and surgical admissions in a secondary hospital in Cameroon: recognition and outcomes

**DOI:** 10.11604/pamj.2025.50.22.31772

**Published:** 2025-01-13

**Authors:** Denis Georges Teuwafeu, Arisha Ayonghe, Ronald Gobina Mbua, Clovis Nkoke, Maimouna Mahamat, Mokake Divine, Gloria Ashuntantang

**Affiliations:** 1 Faculty of Health Sciences, University of Buea, Buea, Cameroon,; 2 Buea Regional Hospital, Buea, Cameroon,; 3 Faculty of Medicine and Biomedical Sciences, University of Yaoundé I, Yaoundé, Cameroon,; 4 Yaoundé General Hospital, Yaoundé, Cameroon

**Keywords:** Acute kidney injury, non-renal medical admissions, surgical admissions, recognition, outcomes

## Abstract

**Introduction:**

there is a paucity of data on the burden of acute kidney injury (AKI) in non-renal medical and surgical admissions where renal function monitoring is not routinely done. This study evaluated the incidence and outcomes of AKI in non-renal medical and surgical admissions at risk of AKI.

**Methods:**

we prospectively assessed non-renal medical and surgical admissions at the Buea Regional Hospital during a 6-week period for AKI risk factors. Consenting participants with AKI risk factors were then screened for AKI using the modified KDIGO (Kidney Disease Improving Global Outcomes) criteria. We excluded patients with a history of Chronic Kidney Disease (CKD), confounders of serum creatinine (e.g. cimetidine, limb amputees), and those without a second serum creatinine value. Modifiable AKI risk factors were corrected and patients with AKI were presented to the nephrologist. Patients were followed up until hospital discharge or death. The outcome measures were the presence of AKI, need and access to dialysis, renal recovery on discharge, for both participants with and without AKI, death, and length of hospital stay.

**Results:**

a total of 165 (41.6% males) participants were included, and six were excluded. The mean (SD) age was 50.7 (17.29) years. Hypertension 43 (26.06%), obesity 28 (16.97%), Human Immunodeficiency Virus (HIV) 25 (15.15%), and diabetes mellitus 22 (13.33%) were the most frequent co-morbid conditions. Sepsis 110 (66.67%) and volume depletion 69 (41.82%) were the most common AKI risk factors. The incidence of AKI was 27.3% (n=45), with 35.6% (n=16) of these in KDIGO AKI stage 3. A total of 4 (8.9%) required dialysis with a 100% access rate. The in-hospital mortality was 6.6% (11/165), with the rate significantly higher in the AKI group (17.78%) compared to the non-AKI group (2.50%) (HR: 2.3, CI: 1.48-2.80, p=0.001). Complications of AKI accounted for 27.27% (3/11) of all causes of death. The median length of hospital stay was longer in the AKI group (11(6-15)) without a statistically significant difference compared to the non-AKI group (8(6-12.5)) (HR: 1.04, CI: 0.99-1.09, p=0.103). Renal recovery on discharge was complete in 62.2% of survivors.

**Conclusion:**

the incidence of AKI is high in non-renal medical and surgical admissions at the Buea Regional Hospital and it is associated with a high mortality.

## Introduction

Acute kidney injury (AKI) which includes acute renal failure is defined as a sudden decline in kidney function (glomerular filtration rate, which results in retention of nitrogenous waste products and disturbances of fluid and electrolyte homeostasis) which can be reversible if detected early [1]. AKI is an increasing public health problem worldwide, associated with adverse outcomes. It is estimated that 13.3 million cases of AKI occur yearly, with 85% occurring in low and middle-income countries [2]. AKI is particularly common in the hospital setting with incidence rates ranging from 22% to as high as 67% in the Intensive Care Unit (ICU) [3]. The rates vary from 16.1% to 20% in medical admissions and 18% to 47% in surgical admissions [4-8]. In hospital admissions, AKI increases the length of hospital stay by 5-7 days, the cost of hospitalization by 2 to 3-fold, and the mortality by 6.5-fold [9]. Apart from these short-term outcomes, AKI increases the risk of Chronic Kidney Disease (CKD) and cardiovascular diseases [10,11]. There is enough evidence to suggest that, late recognition of AKI leads to delay in management leading to increased morbidity and mortality. Deficiencies in AKI care have been reported worldwide. Atiken *et al*. reported a delay in recognition of AKI in 43% of admitted patients in the United Kingdom (UK) and 24% of cases of AKI were unrecognized in another UK study [12,13]. In a recent nationwide survey in China, the in-time recognition of AKI was low in non-renal medical and surgical departments [3]. There is some evidence suggesting that early diagnosis and timely therapeutic strategies may be the cornerstone of future improvement in outcomes [14].

In Low and Middle-Income Countries (LMIC), outcomes of AKI are severe, with 70% of adults needing dialysis, and a mortality of 32% in adults, which rose to 86% when dialysis was needed but not received [1]. The high mortality rate is a consequence of delays in presentation to health facilities, delays in recognition, and delays in management. In addition, limited access to renal replacement therapy (RRT) due to unavailability and unafordability is a major contributor to the abysmal picture. The access to dialysis in sub-Saharan Africa ranges from 21.4% to 72.2% [15-18]. However, most of the epidemiological data on AKI in LMIC in general and Cameroon in particular stems from observational studies in nephrology units and medical units including renal admissions where selected severe cases are more likely with consequent high need for dialysis and mortality [15-17]. In other non-renal medical and surgical admissions where renal function monitoring is not routine, the true incidence may be higher [19]. This study aimed to determine the incidence, characteristics, and outcomes of AKI in non-renal medical and surgical admissions in a government-funded secondary hospital with haemodialysis facilities.

## Methods

**Study design and setting:** this was a 6-week hospital-based prospective cohort study, from March 4^th^ to April 15^th^, conducted in the medical and surgical units of the Buea Regional Hospital (BRH) in the South West Region of Cameroon. It is a referral hospital for the region and a teaching hospital for medical and nursing students. In addition to outpatient services, it has four main in-patient services: medical, surgical, pediatric, and obstetrics and gynecological units. It also hosts the lone hemodialysis unit of the South West Region. The hospital has several clinical specialists: in the medical ward, there are 2 nephrologists, a cardiologist, and a neurologist, who take part in the day-to-day management of hospitalized patients while in the surgical ward, there is 1 surgeon and 1 orthopedist who take part in the day-to-day management of surgical admissions. The medical ward has a capacity of 40 beds and a patient turnover of about 120 patients monthly. The surgical ward has 23 beds with about 40 admissions monthly. The hospital has a South African National Accreditation System (SANAS) accredited functional laboratory, headed by a laboratory scientist.

**Study population:** we included all consenting adult non-renal admissions with at least one AKI risk factor [12]. Patients with a known history of CKD, and confounders of serum creatinine such as patients on cimetidine, on cotrimoxazole, limb amputees, crush injury, and those without a second serum creatinine value were all excluded from this study.

We used Cochran´s formula for calculating sample size, the population (p) of AKI in acute medical admissions in a study that screened for AKI risk factors in the UK by Roberts *et al*. (12.3%) [20]. Therefore, the minimum sample size was 166 participants. The sampling method was consecutive as they were admitted during the period of the study.

**Data collection:** patients with at least one risk factor of AKI were invited to partake in this study and informed consent was obtained. Variables of interest were socio-demographic including age and sex, clinical parameters including working diagnosis, and comorbid conditions. For each patient, the value of creatinine was obtained using the Jaffe kinetic method at admission and the patient was followed up untill discharge or death. For each patient, the creatinine was repeated after 2 days to 5 days and at discharge. Outcomes of interest were the presence of AKI, need and access to dialysis, length of hospital stay, and renal recovery. Those without AKI were followed up to identify de-novo AKI risk factors. Modifiable risk factors were corrected.

**Definition of operational terms:** non-renal admissions were defined as patients admitted for reasons not related to the kidney and not from nephrologist consults. Baseline serum creatinine was the first serum creatinine done on identification of AKI risk factor, or a documented serum creatinine value within 7 days. AKI was defined according to the modified Kidney Disease Improving Global Outcomes (KDIGO) 2012 criteria [21] as an increase or decrease in serum creatinine of 0.3 mg/dl within 48 hours from the baseline. AKI severity was graded using KDIGO 2012 criteria [22].

The need for dialysis was defined as those with indications for dialysis and access to dialysis was defined as those with indications for dialysis that were actually dialyzed. Renal recovery was evaluated at discharge and defined as: 1) complete if normalisation of serum creatinine and; 2) partial recovery if persistence of renal failure without the need of dialysis in those who were receiving dialysis or a decrease in serum creatinine of less than 50% from the value at diagnosis and; 3) no recovery if no decrease in serum creatinine or a decrease of less than 25% or dependency on dialysis.

**Ethical considerations:** ethical approval was obtained from the institutional review board of the University of Buea (2019/898-01/UB/SG/IRB/FHS) and administrative authorizations from the Delegation of Health and BRH. Following a clear explanation of the study in the language best understood by the patient, a consent form was obtained from each patient. The patient was free to withdraw his consent study at any time and no one was sanctioned for not participating. We attributed identification codes to all participants to ensure anonymity throughout the study. We kept the consent forms containing their names separate from the data collection forms. The results of the serum creatinine assay were shared with the participants and the treating physician. All standard procedures during the collection of venous blood samples for serum creatinine assay were respected. This study did not interfere with the procedure of patient care.

**Statistical analysis:** data was analysed using the statistical package for social sciences software, SPSS version 22.0. Quantitative data was summarized using means (standard deviation) and medians (interquartile range) while categorical data was described using numbers and proportions. The median length of hospital stay was compared between the AKI and non-AKI groups using the Mann-Whitney test. Mortality rates in both groups were compared using the Chi-squared test.

## Results

**Figure 1:**
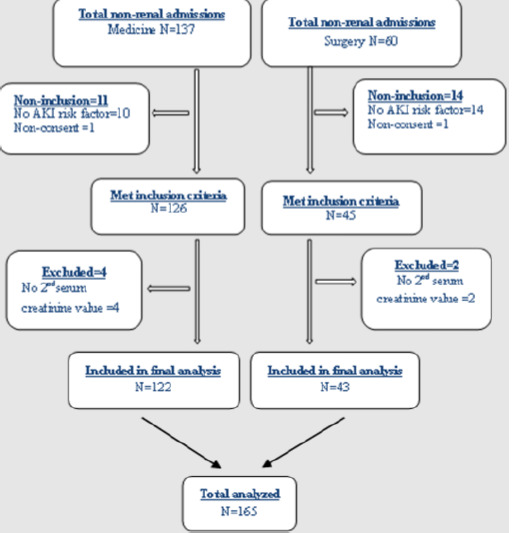
flow diagram of the recruitment of study participants (N=165)

**Figure 2:**
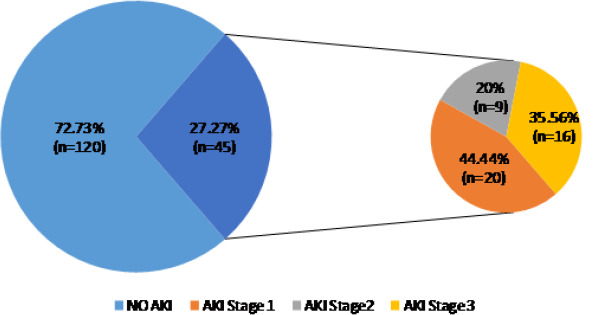
incidence and severity of AKI in the study population

A total of 171 non-renal admissions from the medical and surgical wards were eligible to participate in the study (Figure 1). Six participants were excluded for lack of a second serum creatinine value. Hence, 165 participants were included in the study. The female-to-male ratio was 1.17. The mean (SD) age was 50.7 (17.29) years. Hypertension and obesity were the most frequent co-morbid conditions (Table 1). The main reasons for admission in the medical ward were infections and neurological diseases, with the most frequent pathologies being malaria and cerebrovascular accidents while in the surgical ward, injury, and skin diseases accounted for over 50% of admissions and the most common pathologies were fractures and ulcers (Table 2). The mean (SD) number of AKI risk factors per participant was about three (2.98 ± 1.22). Sepsis 110 (66.67%), volume depletion 69 (41.82%), and use of the renin-angiotensin-aldosterone system (RAAS) blockers 45 (27.77%) age >65 42 (25.45%) were the most common AKI risk factors (Table 3). We found a high incidence of 27.3% of AKI with 35.6% of all cases classified as severe (Figure 2). Of the 45 participants with AKI, 62.2% (n=28) were community-acquired.

**Table 1:** distribution of socio-demographic characteristics and comorbidity of the study population recruited from the medical and surgical wards of Buea Regional Hospital (BRH) according to sex (N=165)

Variable	Total n (%)	Male (n=76) n (%)	Female (n=89) n (%)
Mean age in years (SD)	50.73(17.29)	49.88(16.36)	51.46(18.11)
**Age ranges (years)**			
(18-35)	31(18.79)	14(18.42)	17(19.10)
(35-55)	78(47.27)	42(55.26)	36(40.45)
(55-65)	13(7.88)	3(3.95)	10(11.24)
≥65	43(26.06)	17(22.37)	26(29.21)
**Sources of funding**			
Self	48(29.09)	32(42.11)	16(17.98)
Family	107(64.85)	39(51.32)	68(76.40)
Insurance	10(6.06)	5(6.58)	5(5.62)
**Comorbidities**			
Hypertension	43(26.6)	28(36.84)	15(16.85)
Obesity	28(16.97)	10(13.15)	18(20.37)
HIV	25(15.15)	10(13.15)	15(16.85)
Diabetes mellitus	22(13.33)	12(15.78)	10(11.23)
Heart failure	3(1.82)	2(2.63)	1(1.12)
Malignancy	3(1.82)	2(2.63)	1(1.12)

SD: standard deviation; BRH: Buea Regional Hospital

**Table 2:** participants diagnosis/causes of admission according to the International Classification of Diseases 11^th^ edition (N=165)

Working diagnosis	Number (n)	Frequency (%)
**Medical ward (N=122)**		
**Infectious diseases**		
Malaria	18	14.75
Gastroenteritis	9	7.38
Meningo encephalitis	8	6.56
Extrapulmonary tuberculosis	2	1.64
**Diseases of the nervous system**		
Cerebrovascular accident	20	16.39
Spinal cord compression	11	9.02
**Diseases of the digestive system**		
Upper gastrointestinal bleeding	6	4.92
Liver cirrhosis	3	2.46
**Diseases of the respiratory system**		
Pulmonary tuberculosis	10	8.20
Pneumonia	8	6.56
Chronic obstructive pulmonary disease	2	1.64
**Diseases of the circulatory system**		
Decompensated heart failure	10	8.20
Hypertensive emergency	5	4.09
Decompensated diabetes	7	5.74
**Surgical ward (N=43)**		
**Injury**		
Fracture	13	30.23
Head injury	2	4.65
Hematoma	1	2.33
**Skin disease**		
Ulcer	10	23.26
Cellulitis	3	6.98
Abscess	2	4.65
**Other**		
Intestinal obstruction	4	9.30
Appendicitis/peritonitis	5	11.53
Hernia	2	4.65

**Table 3:** AKI risk factor distribution of participants recruited in the medical and surgical wards of the Buea Regional Hospital (BRH) (N=165)

Variable	Frequency (n)	Percentage (%)
**Socio-demographic**		
Age >65 years	42	25.45
**Comorbidities**		
HIV/AIDS	25	15.15
Diabetes mellitus	22	13.33
Liver failure	5	3.03
Heart failure	3	1.82
Chronic kidney disease (stages 1-4)	3	1.82
Chronic obstructive pulmonary disease	1	0.61
**Drugs that interfere with renal hemodynamic**		
RAAS blockers	45	27.27
NSAIDs	40	24.85
Furosemide	9	5.45
Acute clinical conditions		
Sepsis	110	66.67
Volume depletion	69	41.82
**Nephrotoxic agents**		
Traditional medicine use	41	24.24
Aminoglycosides use	19	11.51
Tenofovir use	15	9.09
Iodinated contrast agents	10	6.06
Chemotherapy/cisplatin use	3	1.82
Perioperative AKI risk factors	15	9.09

AKI: acute kidney injury; HIV/AIDS: Human Immunodeficiency Virus/Acquired Immunodeficiency Syndrome; NSAIDs: non-steroidal anti-inflammatory drugs; RAAS: renin-angiotensin-aldosterone system

**Figure 3:**
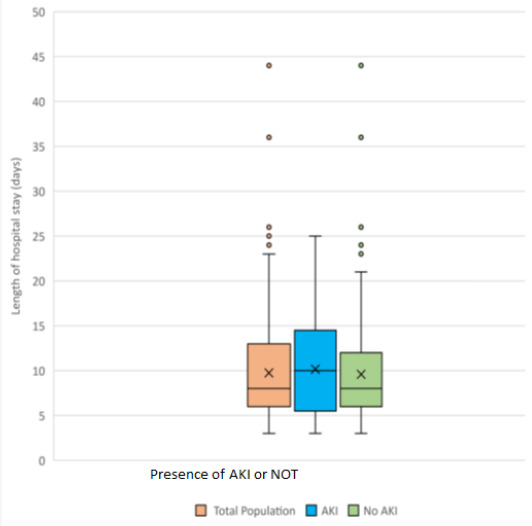
comparison of length of hospital stay between those with and those without AKI

The in-hospital mortality was 6.6% (11/165), with the rate significantly higher in the AKI group (17.78%) compared to the non-AKI group (2.50%) (HR: 2.3, CI: 1.48-2.80, p=0.001). Complications of AKI accounted for 27.27% (3/11) of all causes of death. The median length of hospital stay was longer in the AKI group (11 (6-15)) without a statistically significant difference compared to the non-AKI group (8 (6-12.5)) (HR: 1.04, CI: 0.99-1.09, p=0.103 (Figure 3).

**Figure 4:**
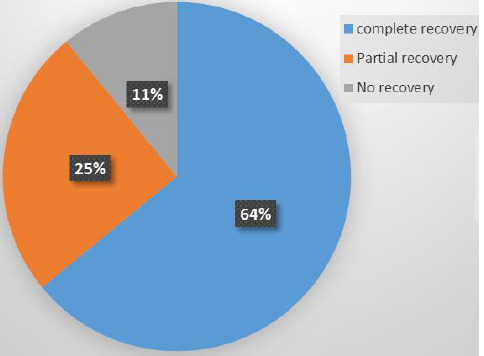
recovery of renal function on discharge

Of the 37 survivors in the AKI group, 23 (62.16%) had complete recovery of renal function on discharge (Figure 4).

## Discussion

In this study, we sought to study the incidence and outcomes of acute kidney injury (AKI) in non-renal medical and surgical admissions in the Buea Regional Hospital. We found a high incidence of 27.3% of AKI, with 35.6% of cases classified as severe. The incidence was significantly higher in non-renal medical admissions, and of the four (8.9%) participants who required dialysis all were dialyzed. We observed a significantly higher mortality rate in the AKI group compared to the non-AKI group (17.8% versus 2.5%, p-value=0.001), while the median length of hospital stay was longer in the AKI group without a statistically significant difference compared to the non-AKI group (11{6-15} versus 8{6-12.5} p=0.103). Renal recovery was complete in 62.2% of survivors in the AKI group.

With the aim of eliminating preventable deaths from AKI by 2025, especially in LMIC, the International Society of Nephrology (ISN) developed the 5Rs that permit timely diagnosis and treatment of potentially reversible AKI for patients [14]. Early recognition of patients at risk for developing AKI is therefore essential to allow early intervention to minimize further renal injury. In this study, a global incidence of 27.3% of AKI was found, with an incidence of 29.5% in the medical unit and 20.9% in the surgical unit. This falls in the range of 22.3% to 35.5% incidence rates described in previous studies from hospitalized patients in Cameroon [15-17]. This finding is in contrast to the pooled worldwide incidence of 21.6% [10]. Lower AKI incidence rates 16.6%-20% were reported among medical admissions, and surgical admissions in other Low- or Middle-Income Country (LMIC) and high-income countries (HIC) [4-8]. The high incidence rates in this study can be explained by the fact that we actively identified and screened non-renal admissions with AKI risk factors. In addition, infectious diseases and sepsis, potent risk factors of AKI accounted for about a third of the reasons for admissions in our study population, increasing the risk of developing AKI. Moreover, 87% of our patients had two or more risk factors, which places them in a cohort of patients at risk of developing AKI [6,20]. Our results are consistent with similar studies in the UK and in the United States, which identified AKI risk factors and routinely screened for AKI, and found incidence rates of 25.55% and 28.8% respectively [7,23]. Roberts *et al*. reported lower incidence rates of 12.3% in patients at risk for community-acquired AKI in acute medical admissions [20].

In contrast to previous reports in sub-Saharan Africa (SSA), we found a low 35.6% incidence of severe AKI (KDIGO stage 3). Reported rates of severe AKI prevalence vary from 48%-77% in LMICs and 41%-47% in HICs [1,24]. In Cameroon, the rate was 59.2% in the same hospital, and 77% in a tertiary hospital [15,17]. Epidemiological studies with a similar methodology to ours revealed lower rates of severe AKI varying from 17.6%-52.5% in the Intensive Care Unit (ICU) [11,18,19]. This lower proportion of severe AKI may be explained by the early recognition of AKI through actively screening for risk factors of AKI, monitoring renal function in those at risk, and correcting risk factors among other measures. Other studies were conducted in renal units where patients are referred when the renal failure is already severe. Our results therefore suggest that systematic identification of patients at risk of AKI, and monitoring their renal function reduces the frequency of severe AKI. This finding is important, especially in LMIC where access to dialysis is limited by cost and availability [24]. Consistent with previous reports in Cameroon and other LMICs, AKI was predominantly community-acquired (62%) in contrast to hospital-acquired AKI reported in HIC [1,24]. Community-acquired AKI accounts for over 70% of cases in adults in LMICs, and 40-50% in HIC [24-26]. Olowu *et al*. reported the prevalence to range from 72.8 to 89.2% in SSA [1].

We noted that 8.9% of patients with AKI required hemodialysis, which is the only modality of renal replacement therapy available at the Buea Regional Hospital. This is much lower compared to the 36% reported in the same hospital and the 50.9% reported in Douala [15,17]. One major explanation for the low rate of dialysis needed in our study is that we identified patients at risk of AKI and routinely screened. As such, AKI cases were detected at an early stage. This reflects the low prevalence of severe AKI as this has been associated with an increased need for dialysis [1]. Our results are similar to Halle *et al*. 10.1% need for dialysis [16]. Higher rates up to 70% were reported in sub-Saharan Africa [1]. The pooled access to dialysis rate has improved from 17% to 47% in the period 2010-2014 [1]. We found a high 100% access rate to dialysis. The access rate in most sub-Saharan countries is dependent on the availability of a dialysis centre and the ability to pay. Our high access rate reflects the availability of a dialysis centre in the hospital, as well as the subsidized cost of hemodialysis in the country. Relatively lower 66.7% and 72.2% access rates were reported in Buea and Douala, respectively due to lack of funds and adequate materials [16,17]. Olowu *et al*. reported a 58% access rate in sub-Saharan Africa [1]. In HIC access rates go as high as 100% because of the insurance coverage which subsidizes funds [24-27].

Despite a better understanding of the pathophysiology of AKI, and the advances in medical techniques, AKI has been shown to independently increase in-hospital mortality rate [21,27-32]. AKI multiplies the risk of death by 6 [28]. The reported in-hospital mortality rate of AKI is high varying from 32% to 44.45% [1,5]. Previous observational studies in Cameroon reported high mortality rates of 10.9%-23.5% [15-17]. In this study, we observed an in-hospital mortality rate of 17.8% in the AKI group, which was significantly higher compared to the non-AKI population (2.5%) but consistent with the findings in other African studies with similar study designs. The ISN Global snapshot reported a mortality rate of 13.3% in Africa, and 12% in LMIC [24]. In some unpublished studies with similar designs in Cameroon. The mortality rate ranges from 23.5% in post-operative AKI, 10.9% in pregnancy-related AKI, and 15.6% in the AKI in the ICU. The lower proportion of severe AKI resulting from early recognition and the excellent access in this study may explain the lower mortality as it allows for prompt management of both risk factors and AKI, and consequentially better outcomes [28].

AKI increases the length of hospital stay by 5 to 7 days with the severity of AKI and the need for dialysis being the main drivers of this increase [7,9,23]. We observed a longer median duration of hospital stay of 11 days in survivors with AKI compared to 8 days in those without AKI although this difference did not attain statistical significance. This may reflect the low need for dialysis rate and the low prevalence of severe (stage 3) AKI. About half of our participants with AKI were at stage 1.

Only 50% of patients requiring dialysis will regain normal renal function within 28 days [33]. We observed a 62.2% achievement of complete renal recovery in survivors at discharge. Higher recovery rates were reported when evaluated at 1 month (73%) in Buea and 3 months (84.2%) in Douala [16,17]. Our low recovery rate can be explained by the period we evaluated recovery which was an average of 8 days and reflects our low rate for dialysis needs. Lower rates of 55% were recorded in Douala at 3 months [15]. The younger age population and lower prevalence of severe AKI, which are associated with better outcomes, can explain our higher rate [24,27]. Complete recovery rates of >60% were reported in Low Income Countries (LIC) [1].

Limitations to this study were: the sample size was less than the minimum expected. However, 95% of all non-renal and surgical admissions with AKI risk factors, were included in the final analysis, so our sample is representative. The risk stratification classification we used was obtained from studies done in other LICs and HICs. It is thus possible that we missed out on locally relevant and specific risk factors, thus missing out on some cases of AKI. We evaluated renal recovery on discharge so long-term outcomes in those with partial or no recovery, such as end-stage kidney disease could not be evaluated. The presence of a dialysis centre in the study setting may have contributed to the high access rates to dialysis.

## Conclusion

We have shown that the incidence of AKI is high amongst non-renal medical and surgical admissions. The frequency of severe AKI and the need for dialysis are low. Although the mortality rate and length of hospital stay are higher in patients who develop AKI, the rates are better than those reported in observational studies in the same centre. About 60% of those who developed AKI completely recovered their renal function on hospital discharge.

### What is known about this topic

- Acute kidney injury (AKI) is particularly common in the hospital setting with incidence rates ranging from 22% to as high as 67% in the ICU;
- The rates vary from 16.1% to 20% in medical admissions and 18% to 47% in surgical admissions;
- In hospital admissions, AKI increases the length of hospital stay by 5-7 days, the cost of hospitalization by 2 to 3-fold, and the mortality by 6.5-fold.

### What this study adds

- The incidence of AKI is high amongst non-renal medical and surgical admissions;
- The frequency of severe AKI and the need for dialysis are low;
- About 60% of those who developed AKI completely recovered their renal function on hospital discharge; the mortality rate and length of hospital stay is higher in patients who develop AKI.
